# Implementation of a novel continuous fetal Doppler (Moyo) improves quality of intrapartum fetal heart rate monitoring in a resource-limited tertiary hospital in Tanzania: An observational study

**DOI:** 10.1371/journal.pone.0205698

**Published:** 2018-10-11

**Authors:** Benjamin A. Kamala, Hege L. Ersdal, Ingvild Dalen, Muzdalifat S. Abeid, Matilda M. Ngarina, Jeffrey M. Perlman, Hussein L. Kidanto

**Affiliations:** 1 Faculty of Health Sciences, University of Stavanger, Stavanger, Norway; 2 Department of Obstetrics and Gynecology, Muhimbili National Hospital, Dar es Salaam, Tanzania; 3 Department of Anaesthesiology and Intensive Care, Stavanger University Hospital, Stavanger, Norway; 4 Department of Research, Stavanger University Hospital, Stavanger, Norway; 5 Department of Obstetrics and Gynecology, Temeke Regional Referral Hospital, Dar es Salaam, Tanzania; 6 School of Medicine, Aga Khan University, Dar es Salaam, Tanzania; 7 Department of Pediatrics, Weill Medical College, New York, New York, United States of America; University of Oxford, UNITED KINGDOM

## Abstract

**Background:**

Intrapartum Fetal Heart Rate (FHR) monitoring is crucial for the early detection of abnormal FHR, facilitating timely obstetric interventions and thus the potential reduction of adverse perinatal outcomes. We explored midwifery practices of intrapartum FHR monitoring pre and post implementation of a novel continuous automatic Doppler device (the Moyo).

**Methodology:**

A pre/post observational study among low-risk pregnancies at a tertiary hospital was conducted from March to December 2016. In the pre-implementation period, intermittent monitoring was conducted with a Pinard stethoscope (March to June 2016, n = 1640 women). In the post-implementation period, Moyo was used for continuous FHR monitoring (July-December 2016, n = 2442 women). The primary outcome was detection of abnormal FHR defined as absent, FHR<120or FHR>160bpm. The secondary outcomes were rates of assessment/documentation of FHR, obstetric time intervals and intrauterine resuscitations. Chi-square test, Fishers exact test, t-test and Mann-Whitney U test were used in bivariate analysis whereas binary and multinomial logistic regression were used for multivariate.

**Results:**

Moyo use was associated with greater detection of abnormal FHR (8.0%) compared with Pinard (1.6%) (*p*<0.001). There were higher rates of non-assessment/documentation of FHR pre- (45.7%) compared to post-implementation (2.2%) (*p*<0.001). At pre-implementation, 8% of deliveries had FHR documented as often as ≤ 60 minutes, compared to 51% post-implementation (*p*<0.001). Implementation of continuous FHR monitoring was associated with a shorter time interval from the last FHR assessment to delivery i.e. median (IQR) of 60 (30,100) to 45 (21,85) minutes (*p*<0.001); and shorter time interval between each FHR assessment i.e. from 150 (86,299) minutes to 60 (41,86) minutes (*p*<0.001). Caesarean section rates increased from 2.6 to 5.4%, and vacuum deliveries from 2.2 to 5.8% (both *p*<0.001). Perinatal outcomes i.e. fresh stillbirths and early neonatal deaths were similar between time periods. The study was limited by both lack of randomization and involvement of low-risk pregnant women with fewer adverse perinatal outcomes than would be expected in a high-risk population.

**Conclusion:**

Implementation of the Moyo device, which continuously measures FHR, was associated with improved quality in FHR monitoring practices and the detection of abnormal FHR. These improvements led to more frequent and timely obstetric responses. Follow-up studies in a high-risk population focused on a more targeted description of the FHR abnormalities and the impact of intrauterine resuscitation is a critical next step in determining the effect on reducing perinatal mortality.

## Introduction

Worldwide 40% of 1.2 million stillbirths are intrapartum-related, i.e., termed fresh stillbirths (FSB) [1]. Some of these deaths are sometimes misclassified as early neonatal deaths (END) [2]. The identification and potential prevention of these FSB has not been addressed in the Sustainable Development Goals (SDG) [3,4]. About 2.6 million babies die annually during the neonatal period, of whom approximately 36% die on the first day, and 73% during the first week of life [5,6]. These neonatal deaths account for approximately 46% of all under-five deaths [6], an increase from the approximately 40% noted in 2000 [7,8] due to decline in mortality in other ages. Nearly a quarter of these neonatal deaths are intrapartum-related and occur mostly in low-resource settings [6,9–11].

Fetal heart rate (FHR) monitoring is crucial for the early screening and identification of existing or impending asphyxia. Studies show that an abnormal FHR detected during labor is associated with intrapartum fetal hypoxia, which may lead to an FSB, END or a live-born infant with variable degrees of hypoxic-ischemic brain injury [2,9,12–15]. Hence, early detection of a hypoxic state is a first step in potentially preventing these important problems. It is estimated that approximately 3 million deaths related to FSB and END could potentially be prevented by equipping and training health workers with tools, i.e., enhanced FHR monitoring capability to enhance the quality of care around the time of birth [12].

Indeed, improved FHR monitoring, coupled with the use of partogram documentation, has the potential to reduce intrapartum-related perinatal deaths [15–17]. Several reports show that appropriate documentation is completed in less than half of all deliveries [16–18], due to competing priorities and shortages of staff [19]. For example, in a tertiary hospital in Zanzibar, the ratio of birth attendant to laboring women was 1:6 [10], far less than the recommended 1:1 ratio for high-risk deliveries [20,21].

Auscultation with a fetal stethoscope, and occasionally with a fetal Doppler, are often the only method of fetal monitoring available in many low-resource settings [22,23]. In high-resource countries, cardiotocograph (CTG) is used, but complexities including high cost and need to continuous electricity supply limit use in low-resource settings [24]. Wyatt et al. theorized that the ideal device for these settings should be affordable and simple to operate [24].

The recent development of a novel strap-on FHR monitor, called Moyo, has facilitated a more rapid identification of the FHR and may be a breakthrough in identifying fetuses at high-risk of intrapartum hypoxia-ischemia. Although the reliability of the device is difficult to ascertain, it is noteworthy that in a recent qualitative assessment among mothers in Tanzania, it was noted that Moyo was the preferred device to use. This likely reflects the maternal-midwife interactive nature of the device, as well as the ability of the mother to hear fetal heart sounds providing “reassurance” of her fetus wellbeing [25].

We hypothesized that continuous FHR monitoring device will facilitate detection of abnormal FHR and timely interventions. The primary objective of the present study was to compare continuous FHR monitoring during labor using the Moyo device with prior intermittent FHR monitoring using a Pinard stethoscope for the detection of FHR abnormalities defined as absent, FHR<120 or FHR>160bpm in a resource-constrained tertiary hospital. Secondary outcomes were subsequent obstetric interventions, partogram documentation, frequency of newborn resuscitations, and the effect on perinatal outcomes.

## Methods

### Study design

A pre/post observational analytical study among low-risk pregnancies was conducted from March through December 2016 at Temeke Regional Referral Hospital in Dar es Salaam. In the pre-intervention period of 3 months (March to June), Pinard stethoscopes were used intermittently, and in the post-intervention period of 5 months (July to December), Moyo devices were used for continuous monitoring of FHR during labor.

### The intervention

**Fig 1:**
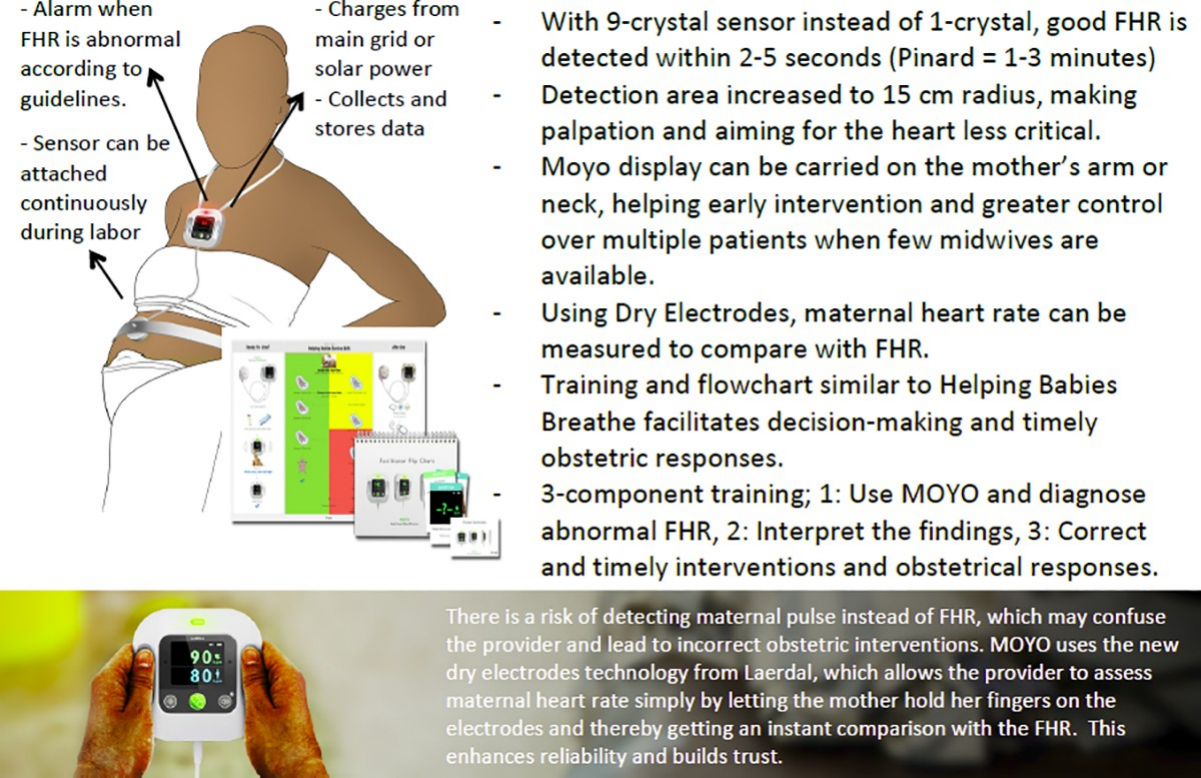
Moyo–The new continuous fetal heart rate monitor (Laerdal Global Health). FHR abnormalities defined as absent, FHR<120 or FHR>160bpm).

The Moyo (Laerdal Global Health, Stavanger, Norway) device is a novel strap-on FHR monitor equipped with a battery, containing a nine-crystal Doppler ultrasound sensor which facilitates the rapid identification of FHR (Fig 1). It can be used in either continuous or intermittent mode. The detection area reaches about 15 cm in radius, which makes palpation and aiming for heart beats less critical. Using a set of dry electrodes, maternal heart rate can be differentiated from FHR. The Moyo displays a 30-minute historical graph of FHR, as well as an audio-visual alarm which alerts the midwife every time there is an abnormal FHR or undetected FHR lasting for more than three minutes and continued alarming until something was done to correct the abnormality. We collected data on an intervention by the midwife following the initial Moyo alarm but not on subsequent alerts. A training flowchart is also provided to facilitate decision-making and timely responses.

### Study setting

This study was conducted at Temeke, a referral hospital located in Dar Es Salaam, Tanzania. Temeke municipality has a population of about 2 million people [26]. The municipality has about 135 health facilities referring complicated cases to Temeke for advanced care. The hospital has about 30–60 deliveries per day (more than 12 000 per year). Its labor room has 18 beds and a general operating theatre is used for obstetric and other surgical cases. The obstetrics unit has two qualified obstetricians, 12 general doctors, 25 nurse-midwives, five medical attendants and a varying number of rotating intern medical doctors and nurses who perform deliveries. Nurses have three shifts per day with an average of three nurses and one medical attendant per shift. Doctors have two shifts with one medical doctor and two interns during the day and night shifts, respectively. Some emergency cases are referred to Muhimbili National Hospital.

### Training

Study training, focusing on the standard operating procedures for using the Moyo, was conducted in February 2016. The study investigators (MAS, MMN, BAK) trained midwives (*n* = 25) and doctors (*n* = 12) from the labor ward for one day. For both study periods i.e. pre- and post-implementation, training included FHR monitoring during labor (normal FHR range, i.e., 120 to 160 bpm) and the management of an abnormal FHR defined as absent, FHR<120 or FHR>160bpm). The criteria for monitoring were established and included the monitoring and documentation of the FHR every 30 minutes in the first stage of labor, every 15 minutes in the second stage, and immediately after every contraction, as per WHO and other international guidelines [27,28]. Refresher training sessions were conducted monthly to accommodate incoming healthcare workers. Research nurses (2 per shift) were trained separately on the research protocol and data collection to ensure the accuracy and completeness of the data. They observed deliveries in the labor room and followed admissions into neonatal units in shifts.

### Study procedures

During the pre-implementation period, upon admission of the eligible women in the labor ward, a written consent was sought. FHR was to be monitored intermittently by auscultation using a Pinard stethoscope every 30 minutes in the first stage of labor, every 15 minutes in the second stage, and immediately after every contraction. A midwife auscultated the FHR for a complete minute with the Pinard stethoscope. Only baseline FHR was recorded in this study. The Pinard is unable to delineate either decelerations or accelerations. FHR was recorded as abnormal if the FHR was absent or FHR<120 or FHR>160bpm.

During the implementation period, eligible woman admitted to the labor ward was given initial information about the Moyo by the nurse midwife. For those who consented, a Moyo was strapped on for continuous FHR monitoring. The midwife would then continue with her routine activities but also periodically (every 30 minutes) revisit the woman to check and record FHR reading from the Moyo monitor, or when the alarm for abnormal FHR was activated. Similarly, FHR was recorded as abnormal if there was absent heart rate or FHR<120 or FHR>160bpm from the Moyo monitor. The Moyo device was strapped on to the mother until the end of the second stage of labor or just before the commencement of a caesarean section. During both periods, the midwives were supposed to document the FHR in the partogram.

Research nurses recorded the frequency of the partogram and FHR documentation, the intrapartum management of different events (stopping oxytocin, giving intravenous fluids, changing mother’s position) and perinatal outcomes on the data collection form.

### Study population

The study population included every low-risk woman admitted in labor. Exclusions included those scheduled for elective cesarean section, twin pregnancies, women with abnormal FHR on admission i.e. absent; FHR<120 or FHR>160bpm, critically ill patients or with no measurements of FHR on admission, and admission in the second stage of labor coupled with full cervical dilatation.

### Sample size

At Temeke, historical data showed that, when using available fetal auscultation, i.e., a Pinard stethoscope, abnormal FHR was detected in approximately 2.0% of all low-risk deliveries. Assuming an increase in detection rate to at least 5% with the Moyo device, we planned the study to include a minimum of 890 (total 1780) cases pre- and post-implementation, which would give us 90% power with alpha level of 0.05. This sample size was assumed to be reached within a study period of totally 4 months (2 months pre- and 2 months post-implementation), however, due to delays in implementation and to account for missing data, the study period was extended to totally 7.5 months.

### Data collection

Data were collected using a data collection form, containing background characteristics which included maternal age (categorized as < 20, 20–35 and > 35 years), education level (primary, secondary and post-secondary training), marital status (married or single), antenatal care (ANC) attendance (none, 1–3 and > 3 visits), parity (nulliparous, 1–3 and > 3 deliveries), and gestational age (GA, in weeks) which was later dichotomized into preterm and term; all of these variables were extracted from the women’s ANC cards on admission. The recorded labor and delivery variables included source of admission (home, referral or inpatient), presentation of the baby (cephalic or breach), and mode of delivery (normal vaginal, vacuum delivery and caesarean section). Intrauterine resuscitation performed after the detection of abnormal FHR were recorded, and included change of maternal position, discontinuing oxytocin, giving intravenous fluids and oxygen administration. Time intervals included labor ward admission to delivery, last FHR to delivery, and intervals between FHR monitoring.

### Outcome measures

Outcome measures included abnormal FHR detection i.e. absent, FHR<120 or FHR>160bpm, mode of delivery, Apgar score at 5 minutes (low if the score was < 7), resuscitation (stimulation, suction and ventilation), FSB, admissions to neonatal unit, END at 24 hours, and composite perinatal mortality (FSB and END).

### Data management and analysis

The collected data were crosschecked for accuracy and completeness by the investigators before entry. Trained data clerks conducted double-entry of the verified data. Data consistency was checked, and mismatched cases were retrieved and corrected accordingly before analysis. Data analysis was conducted using SPSS (IBM SPSS Statistics for Windows, Version 23.0. Armonk, NY: IBM Corp).

Mean (SD), median (IQR) and proportion were used for descriptive statistics of background variables and outcomes. Pearson’s Chi-square and Fisher’s exact tests were used to test for proportion differences. *T*-test and Mann-Whitney *U* test were used to compare group mean and median respectively. Binary and multinomial logistic regression analyses were used to compare outcome variables pre- and post-implementation of the Moyo. We present unadjusted and adjusted odds ratio (AOR) with 95% confidence intervals (95%CI). STROBE was used as reporting guideline of this study.

### Ethical clearance

This study was part of the Safer Births project, certified by both the National Institute of Medical Research in Tanzania (NIMR/HQ/R.8a/Vol. IX/1434) and the Regional Committee for Medical and Health Research Ethics, Western Norway (REK Vest). Permission to publish was granted by NIMR (NIMR/HQ/P.12 VOL. XXIV/15). Local permission was sought from Temeke Municipal Council. In the labor ward, participants were informed about the study and provided written consent if they agreed to participate. Routine clinical performance and patient information was recorded using confidential codes and these were kept in a safe and secure place by the investigators. Research staff were trained on maintaining confidentiality and signed a confidentiality agreement.

## Results

**Fig 2:**
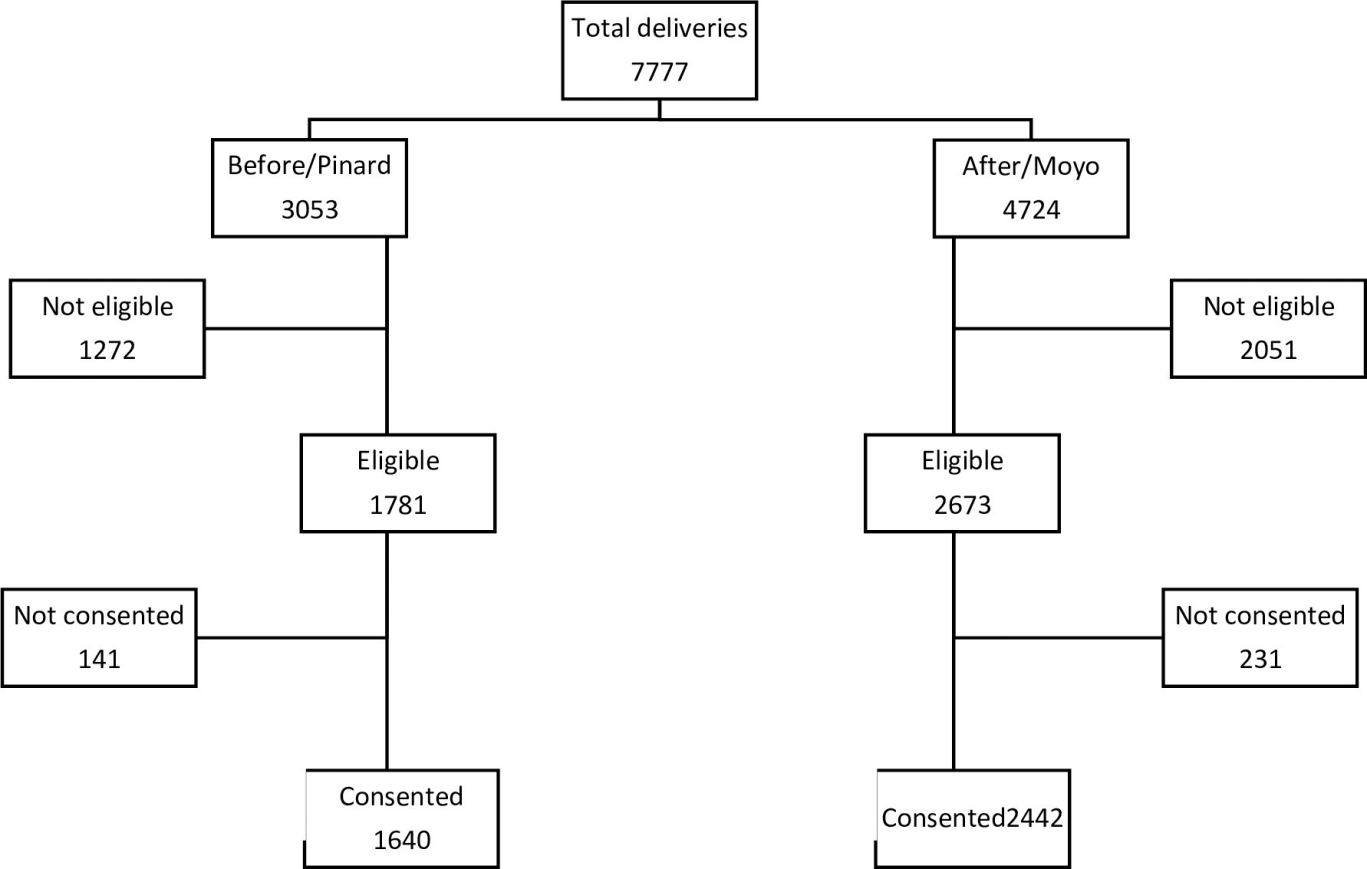
Flow chart of the study population.

During the study period, 7777 deliveries were recorded at the hospital (Fig 2), 3053 pre- and 4724 post-implementations of Moyo. Pre-implementation 1781 women were eligible and 1640 (92%) consented to participate. Post-implementation 2673 women were eligible and 2442 consented to participate (91.3%). Main reasons for exclusion included an abnormal FHR on admission, mothers scheduled for elective caesarean section, and those who presented with full cervical dilatation on admission.

The maternal and obstetric characteristics of the women included in both study periods are shown in Table 1. There were more primigravid mothers, more married women, mothers with a higher educational level, infants of a higher gestational age, and less premature infants (all *p*<0.001) post- versus pre-implementation.

**Table 1:** Baseline maternal and obstetric characteristics of women admitted in the labor ward at temeke hospital pre- and post-implementation of a continuous automatic Doppler (Moyo) from March to December 2016.

Maternal/Obstetrics characteristics		Pre-implementation; Pinard (N = 1640)	Post-implementation; Moyo (N = 2442)
Age (years)	(Mean ± SD)	25.7±6.1	25.4±6.0
	<20	267 (16.3)	394 (16.1)
	20–35	1234 (75.2)	1859 (76.1)
	>35	134 (8.5)	189 (7.7)
Parity	Primigravida	622 (37.9)	1104 (46.9)
	2–4	877 (53.5)	1145 (46.9)
	Grand multiparity	141 (8.6)	193 (7.9)
Source of admission	Home	1050 (64.0)	2025 (82.9)
	Inpatient/Referrals	590 (36.0)	417 (17.1)
Marital status	Married	1243 (75.8)	2074 (84.9)
	Single/cohabiting	397 (24.8)	368 (15.1)
Antenatal visits	None	37 (2.3)	37 (1.5)
	1–3	652 (39.8)	941 (38.5)
	>3	951 (58.0)	1464 (60.0)
Education	Primary	1131 (69.0)	1908 (78.1)
	Secondary and above	509 (31.0)	534 (21.9)
Gestation age (weeks)	(Mean ± SD)	38.4±2.0	38.9±1.7
	Preterm	45 (2.7)	39 (1.6)
	Term	1595 (97.3)	2403 (98.4)
Cervical dilatation (cm)	(Mean ± SD)	6.3±1.5	6.2±1.5
Presentation	Cephalic	1630 (99.4)	2413 (98.8)
	Breech	10 (0.6)	29 (1.2)
*HCW attending delivery	Doctor	86 (5.2)	135 (5.5)
	Nurse/midwife	1554 (94.8)	2307(94.5)

*HCW: Healthcare worker

**Fig 3:**
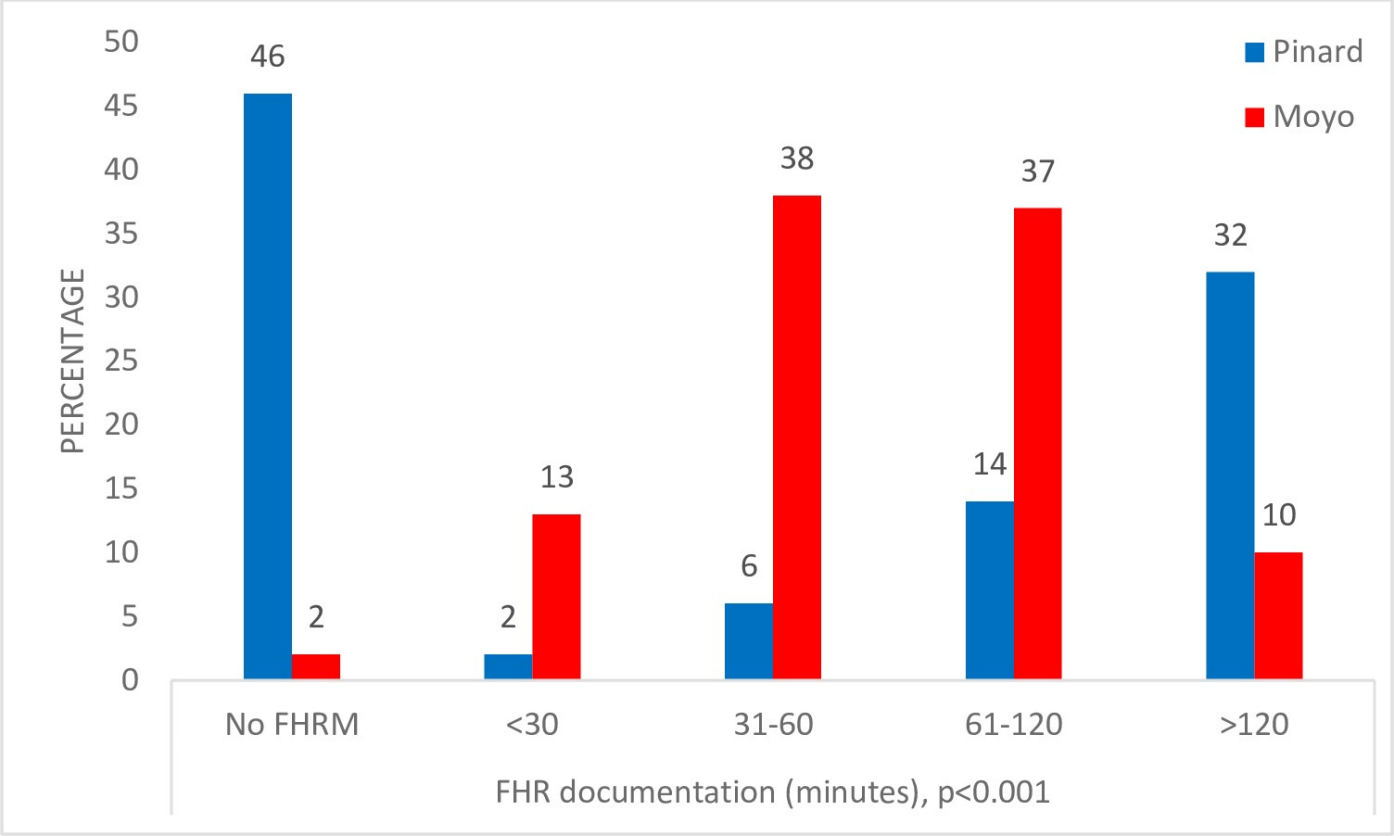
Frequency of Fetal Heart Rate monitoring and documentation post-implementation vs pre-implementation of the Moyo.

Fig 3 shows the frequency of FHR monitoring pre- and post-implementation of the Moyo. During the post-implementation period, 2389/2442 (98%) of the women had the FHR monitored and documented in the partogram compared with the pre-implementation period, which was 890/1640 (54%) of the women (*p*<0.001). Overall, the frequency of the FHR monitoring was higher in post-implementation compared to pre-implementation (*p*<0.001). Post-implementation, 13% of the women were documented every < 30 minutes compared to 2% pre-implementation. Approximately 38% of the mothers had their FHR documented every 30–60 minutes in the post-implementation period compared to 6% pre-implementation. Furthermore, post-implementation, 37% and 10% of the mothers had FHR documented every 61–120 and >120 minutes compared to 14% and 32% pre-implementation, respectively (all p<0.001).

An increased proportion of women received intrauterine resuscitation (i.e., change of mother’s position, stopping oxytocin, starting IV fluids and oxygen administration) post-implementation. Specifically, oxytocin was discontinued in 2.4% as compared to 0.42%; changing position and initiating IV fluids increased to 5.5% from 0.06% and to 6.5% from 0.5%, respectively (all *p*<0.001).

Table 2 shows the proportions, unadjusted and adjusted comparisons of different labor, delivery and perinatal outcomes between the two time-periods. Women had 45 times higher odds of having the FHR monitored and documented post-implementation (AOR 45; 95% CI 34.4–62.5) (*p*< 0.001). An abnormal FHR detection had almost 7 times higher odds of being detected post-implementation (AOR 6.90, 95%CI 3.89–12.24). A caesarean delivery was 5.7 times and a vacuum extraction 3.8 times higher odds post- versus pre-implementation (both *p*< 0.001). Overall, infants had higher odds of receiving any form of resuscitation post-implementation (p < 0.001). More specifically, a lower proportion of babies were stimulated post- versus pre-implementation (11.3% vs 14.8%, *p* = 0.001), whereas a higher proportion received bag mask ventilation post- compared to pre-implementation (5.0% vs 2.6%, *p*<0.001). More babies were admitted to a neonatal area following birth and at 24-hours post-delivery during the post- compared to pre-implementation period (p = 0.001). Perinatal mortality did not differ between the two time periods.

**Table 2:** Proportions, unadjusted and adjusted comparison of FHR documentation practices and outcomes post vs. pre-implementation of strap-on automatic Doppler (Moyo).

Variable	Values	Pre-implementation; Pinard (N = 1640)	Post-implementation; Moyo (N = 2442)	Unadjusted OR (Moyo vs. Pinard)	p-value	Adjusted OR (Pinard vs. Moyo)**	p-value
FHR monitoring during labor	Yes	890 (54.3)	2389 (97.8)	38.46 (28.57–50.0)	<0.001	45.45 (34.4–62.5)	<0.001
	No	750 (45.7)	53 (2.2)	1		1	
FHR during labor*	Normal	876 (98.4)	2198 (92.0)	1		1	
	Abnormal***	14 (1.6)	191 (8.0)	5.44 (3.14–9.41)	<0.001	6.90 (3.89–12.24)	<0.001
Mode of delivery	Normal (SVD)	1561 (95.2)	2167 (88.7)	1		1	
	Caesarean Section	43 (2.6)	133 (5.4)	2.23 (1.57–3.16)	<0.001	5.79 (3.34–10.01)	<0.001
	Vacuum	36 (2.2)	142 (5.8)	2.84 (1.96–4.12)	<0.001	3.851 (2.54–5.83)	<0.001
Received resuscitation	Yes	321 (19.6)	297 (12.2)	0.57 (0.48–0.68)	<0.001	0.63 (0.52–0.75)	<0.001
Stimulation	Yes	242 (14.8)	276 (11.3)	0.74 (0.61–0.89)	0.001	0.86 (0.71–1.06)	0.14
Suction	Yes	210 (12.8)	298 (12.2)	0.95 (0.78–1.14)	0.57	0.99 (0.81–1.22)	0.96
Ventilation attempted	Yes	43 (2.6)	122 (5.0)	1.95 (1.37–2.78)	<0.001	2.28 (1.57–3.30)	<0.001
Apgar score at 5 minutes	<7	25 (1.5)	51 (2.10)	1.58 (0.95–2.64)	0.19	1.58 (0.95–2.64)	0.07
Birth outcomes	Normal	1586 (96.8)	2327 (95.3)	1		1	
	Admitted to neonatal unit	47 (2.9)	107 (4.4)	1.55 (1.09–2.19)	0.014	1.71 (1.18–2.47)	0.005
	Fresh Stillbirths	7 (0.42)	8 (0.33)	0.78 (0.28–2.15)	0.630	0.90 (0.30–2.63)	0.85
Neonatal outcomes 24-hours	Normal	1603 (98.0)	2353 (96.7)	1		1	
	Admitted to neonatal unit	27 (1.7)	74 (3.0)	1.87 (1.19–2.91)	0.006	2.11 (1.33–3.38)	0.002
	END	5 (0.3)	7 (0.3)	0.95 (0.30–3.01)	0.940	0.99 0.29–3.30)	0.97
	Perinatal deaths (FSB+END)	12 (0.7)	15 (0.6)	0.98 (0.43–2.17)	0.958	0.73 (0.31–1.72)	0.47

SVD = Spontaneous vaginal delivery, FSB = Fresh Stillbirths, END = Early neonatal deaths, AOR = Adjusted Odds Ratio

*Only those who were monitored are included in the denominator

**Adjusted for baseline imbalances

*** absent, FHR<120 or FHR>160bpm

There were more caesarean sections (5.4%) post- compared to pre-implementation (2.6%) (p <0.001). The primary indications for caesarean section were: fetal distress, 48% vs 35%; obstructed labour, 9% vs 14%; prolonged labor, 23% vs 40%; and previous CS, 12% vs 9%, in post- versus pre-implementation periods, respectively (*p* = 0.349).

Table 3 shows the median (IQR) time intervals comparing those who had at least one FHR assessed and documented post- versus pre-implementation of the Moyo. The median time interval from admission to delivery was 212 compared to 225 minutes (*p* = 0.002), the median time interval from the last FHR assessment to delivery was 45 versus 60 minutes (*p*<0.001), and the median time interval between FHR documentation in the partogram was every 60 versus every 150 minutes (*p*<0.001), post- versus pre-implementation, respectively. There was no significant difference in time interval from either admission to detection of abnormal FHR or from abnormal FHR detection to delivery.

**Table 3:** Comparison of different median time intervals pre- and post-implementation of the Moyo at Temeke*.

*Time intervals (q1*, *q3)*	*Pre-implementation* *Pinard*	*Post-implementation* *Moyo*	P-value*
Admission to delivery	**(*n* = 1640)**	**(*n* = 2442)**	
	225 (130, 387)	212 (117, 355)	0.002
Admission to Abnormal FHR** detection	*n* = 14	*n* = 191	
	230 (120, 630)	138 (65, 302)	0.184
Last FHR assessment/ documentation to delivery	*n* = 890	*n* = 2389	
	60 (30, 100)	45 (21, 85)	<0.001
Between FHR assessment/ documentation	*n* = 890	*n* = 890	
	150 (86, 299)	60 (41, 86)	<0.001
Abnormal FHR to delivery	*n* = 14	*n* = 191	
	28 (19, 57)	43 (23, 80)	0.255

q1 25^th^ percentile and q3 75^th^ percentile

*Mann-Whitney U test, FHR = Fetal Heart Rate

**absent, FHR<120or FHR>160bpm

## Discussion

The findings in this study demonstrate that implementation of continuous FHR monitoring using a novel Moyo device, was associated with a 6.90-fold increased detection of abnormal FHR i.e. absent, FHR<120 or FHR>160bpm, markedly improved intrapartum FHR monitoring practices, enhanced documentation on the partogram, a reduced time interval from the last FHR assessment to delivery and was coupled with more intrauterine resuscitations. A cesarean section was 5.7-fold higher odds, and a vacuum extraction delivery 3.8-fold higher odds post-implementation. Overall, the need for resuscitation interventions was less post-implementation, however, more babies received bag mask ventilation during the latter period. There were no differences in FSB and END, but there were more admissions to the neonatal unit following delivery and at 24-hours during the post-implementation period.

Adherence to standard clinical practice of FHR monitoring, especially in low income countries, has been persistently inadequate, which likely has contributed to the unchanged rates of FSB and END over time. In order to facilitate FHR monitoring as well as partograph documentation in accordance with international guidelines, strategies have focused on augmenting human resources, pre- and in-service continued training, as well as enhancing supportive supervision [29]. Further studies have addressed poor midwives attitudes, as well as lack of confidence and skills, as additional important factors contributing to suboptimal FHR monitoring [29–31]. Using the Moyo device, the ability of the midwife to identify an abnormal FHR was improved in two ways. First, by visually documenting the details of abnormal FHR in real-time, via the 30-minutes histogram review of the tracing. Second, via activation of an alarm, if the FHR abnormality was of a three-minutes duration. This latter feature allowed the midwife to monitor several mothers simultaneously, which is a major benefit of this device. This translated into improved FHR monitoring practices including timely responses, such as reduced time to the detection of an abnormal FHR following admission, shorter times from the last FHR measurement to delivery as well as shorter overall duration of labor. This is consistent with previous studies showing that improved fetal surveillance was associated with reduction of labor time [32]. In addition, the midwife was then able to respond to the abnormal FHR, by implementing intrauterine resuscitation attempts more frequently, in efforts to reduce intrapartum hypoxia/ischemia as documented earlier [33]. These cumulative findings indicate that by providing continuous FHR monitoring, coupled with an audible alarm system, a significant improvement in midwifery standards and quality of care delivered during the intrapartum period, in accordance with international guidelines, is possible [27,28].

The finding of low FHR monitoring documentation (54%) in the pre-implementation period is consistent with previous studies in Nepal, Ethiopia, and Ghana, where the rates were as low as 20%, 30% and 55%, respectively [18,30,34]. In these countries, the most frequently used device was the Pinard stethoscope. Health care workers with demanding workloads are highly likely to miss important changes in fetal condition with intermittent FHR monitoring [24,35]. The improvements shown in FHR monitoring, and adherence to the partogram in this study, are likely due to the user-friendly features of the Moyo device, which enables the midwife to attend to several patients concurrently, with minimal interruption of routine duties. Despite improved rates, the documentation of FHR monitoring frequencies of < 30 min were still low (13%) with continuous monitoring as compared to the available guidelines [36]. This low frequency of documentation was also reported when using intermittent auscultation FHR monitoring in the high-resource setting (48%) where the midwife-to-patient ratio was nearly 1:1, indicating that other factors may be contributing to this suboptimal documentation [29].

In this study, we noted an increased rate of caesarean section deliveries from 2.6 to 5.4%, presumably in response to the abnormal FHR. This rate is similar with a worldwide population‐based ecological study (2012) that reported an overall caesarean rate of 5.2% in low income countries [37]. The World Health Organization (WHO) suggests that a rate of between 10 and 15% at a population level, may reflect optimal intrapartum care, provided decisions are based on balancing the risks and benefits of this intervention [38,39].

It is noteworthy that while the overall need for resuscitation decreased post continuous monitoring, the number of babies receiving bag mask ventilation increased. This incongruous finding may be due to several interrelated factors. First, the higher incidence of an abnormal FHR may have reflected an intrapartum hypoxia/ischemia state, with resultant respiratory depression upon delivery. Second, the caesarean deliveries were invariably performed under general anesthesia and depending on the duration between initiation of anesthesia and delivery, this may have resulted in the initial respiratory depression, particularly in the setting of an abnormal FHR. There were no differences in FSB and END likely reflecting a low occurrence of these morbidities in this low-risk population. An explanation for the increased number of admissions post-implementation to a newborn area is not entirely clear. However, more mothers underwent caesarean section, invariably under general anesthesia post-implementation. In this setting, mothers are usually separated from their newborns for 24 hours after the caesarean section. In addition, some of these neonates were admitted for observation following bag mask ventilation, and/or were waiting for their mothers to recover from surgery.

### Limitations

There were several limitations. First, this was a pre- and post-implementation study design, hence there was no randomization. However, we consider the time difference between the pre- and post-implementation period of too short a duration for factors other than the intervention to cause the improvements. Furthermore, there were no observed systemic changes that might have led to improved FHR monitoring. Second, although imbalances in baseline characteristics were observed, these were adjusted in the regression analysis to remove potential confounding effects and improve the precision of the effect measures estimates. Third, the study involved only low-risk pregnancies with fewer adverse perinatal outcomes than would have been expected in the overall population. Fourth, some health workers might have failed to complete the partogram, even if FHR measurements were taken, leading to a misclassification as being non-documented. Fifth, only baseline FHR abnormalities i.e. absent, FHR<120bpm or FHR>160bpm were recorded while early, late decelerations or rapid accelerations were not addressed in this study. Unfortunately, the time from alert to response was not collected in this study. Moreover, we did not collect data on when there was an alert without a response. This important question will be included in future prospective studies in the high-risk population.

### Conclusion

Implementation of the Moyo device, which continuously measures FHR, was associated with improvement in the quality of FHR monitoring practices, and the detection of abnormal FHR (absent; FHR<120bpm or FHR>160bpm) in the resource-constrained setting. These improvements led to more frequent and timely obstetric responses. Follow-up studies in the high-risk population, focused on a more targeted description of the FHR abnormality, including the duration, recurrence, and the relation to uterine contractions, as well as the impact of intrauterine resuscitation on the FHR abnormalities, is a critical next step in determining the impact on reducing perinatal mortality.
